# Clonal Integration of *Fragaria orientalis* in Reciprocal and Coincident Patchiness Resources: Cost-Benefit Analysis

**DOI:** 10.1371/journal.pone.0080623

**Published:** 2013-11-12

**Authors:** Yunchun Zhang, Qiaoying Zhang

**Affiliations:** 1 Department of Botany, Institute of Agricultural and Environmental Sciences, Estonian University of Life Sciences, Tartu, Estonia; 2 Qilu University of Technology, Jinan, Shandong Province, P. R. China; 3 Department of Botany, Institute of Ecology and Earth Sciences, University of Tartu, Tartu, Estonia; Beijing Forestry University, China

## Abstract

Clonal growth allows plants to spread horizontally and to experience different levels of resources. If ramets remain physiologically integrated, clonal plants can reciprocally translocate resources between ramets in heterogeneous environments. But little is known about the interaction between benefits of clonal integration and patterns of resource heterogeneity in different patches, i.e., coincident patchiness or reciprocal patchiness. We hypothesized that clonal integration will show different effects on ramets in different patches and more benefit to ramets under reciprocal patchiness than to those under coincident patchiness, as well as that the benefit from clonal integration is affected by the position of proximal and distal ramets under reciprocal or coincident patchiness. A pot experiment was conducted with clonal fragments consisting of two interconnected ramets (proximal and distal ramet) of *Fragaria orientalis*. In the experiment, proximal and distal ramets were grown in high or low availability of resources, i.e., light and water. Resource limitation was applied either simultaneously to both ramets of a clonal fragment (coincident resource limitation) or separately to different ramets of the same clonal fragment (reciprocal resource limitation). Half of the clonal fragments were connected while the other half were severed. From the experiment, clonal fragments growing under coincident resource limitation accumulated more biomass than those under reciprocal resource limitation. Based on a cost-benefit analysis, the support from proximal ramets to distal ramets was stronger than that from distal ramets to proximal ramets. Through division of labour, clonal fragments of *F. orientalis* benefited more in reciprocal patchiness than in coincident patchiness. While considering biomass accumulation and ramets production, coincident patchiness were more favourable to clonal plant *F. orientalis*.

## Introduction

In natural habitats, essential resources for plant survival, growth and reproduction, such as light and water, are often patchily distributed in space and time [1,2]. Resource heterogeneity occurs even at scales relevant to plant individuals [3,4] and plant parts. Such fine-scale spatial resource heterogeneity affects many ecologically important processes and phenomena, which can range from responses of community [5-9], to populations [10-14], to individuals or parts of individuals [2,15]. 

Clonal plants, especially those with long spacers between ramets, can potentially respond to resource heterogeneity on the between plants scale by enhancing fitness-relevant processes such as resource uptake, clonal expansion and offspring establishment in heterogeneous habitats [4,16]. Clonal plants spread horizontally within their habitat by means of stolons or rhizomes, and establish ramets in patches that may differ in resource supply [17-20]. Connected ramets of the same clone may coordinate their plastic responses to contrasting resource levels and share acquired resources [21-24]. Connections between ramets allow for translocation of resources from source-sites to sink-sites within the clone, which is usually referred to as clonal integration [25,26]. Physiological integration and plasticity of clonal plants have been considered to be adaptive and able to enhance the genet performance of clonal plants, particularly in heterogeneous environments, where their interconnected ramets often grow in different microhabitats [27,28]. However, the benefits of intraclonal resource translocation vary between different levels of resource contrast [21,29,30], while other studies have shown that under some source heterogeneity situations, clonal integration may result in lower fitness, and clonal plants may cease to support dependent ramets [31-33]. So the fitness of clonal plants partly depends on the level of source contrast and the complex of source heterogeneity. 

Clonal plants are often found in scrublands or shrublands, where light availability is lower and water availability is higher than in the immediately surrounding area. This kind of negative association between different resources is called reciprocal patchiness [34]. Clonal plants also frequently inhabit grasslands, where small gaps opened by disturbance or mortality have higher availability of light and water than the surrounding sward [35]. This kind of positive association between different resources is called coincident patchiness [34]. In reciprocal patchiness environments some clonal plant species are known to show functional specialisation by capturing locally abundant resources and exchanging them among ramets through physiological integration (division of labour) [36,37]. Such division of labour between spatially separated units of modular systems conforms to space-economy and economic geography disciplines, which mainly address the problem of maximizing production and profits when resources are restricted and unevenly distributed in space [36,38]. In environments with different essential resources unevenly distributed in space, division of labour is likely to give benefits in terms of whole-system performance by reducing the number of tasks for ramets through efficiency increase [38]. The benefits of division of labour to enhance resource capture of clonal plants and thereby to increase their performance in heterogeneous habitats are identified in many previous studies [37-40]. While, in coincident patchiness environments clonal plants may cease or lower their support to dependent ramets to avoid lowering fitness because of the cost of this unilateral clonal integration [31-33,41]. So the integration between ramets of clonal plants would be more extensive under reciprocal patchiness than under coincident patchiness.

It’s possible to find that both proximal and distal ramets of a clonal fragment locate in the same patchiness, or in different patchiness, separately. At the same time, it can be found that proximal ramets are in rich resource while distal ramets are in poor resource, or inverse (integration direction). Studying clonal plants in complex habitats can help us to understand the role of fine-scale resource heterogeneity in clonal ecology. So far, only one research study has been done on the benefits of clonal integration under either reciprocal or coincident patchiness of above-ground and below-ground resources [34]. However, the fragments of the clonal plants in the experiment were in different total resource levels between the treatments, which may affect the cost-benefit analysis of clonal integration. While in our experiment, we put the fragments of *Fragaria orientalis* in the same total resources to analyse the cost-benefit of integration under heterogeneity, as well as cost-benefit of direction of resource transportation under heterogeneity for the first time. We predicted that 1) effects of clonal integration on the performance of clonal plant *F. orientalis* can differ depending on whether connected ramets experience reciprocal or coincident patchiness of above- and below-ground resources; 2) the integration between ramets of *F. orientalis* would get more benefit under reciprocal patchiness than under coincident patchiness, and 3) the performance of clonal fragments is affected by the integration direction under reciprocal or coincident patchiness.

## Materials and Methods

### Plants and experimental design

*Fragaria orientalis* (Rosaceae) is a stoloniferous, perennial herb which is widely distributed throughout Korea, Mongolia, Eastern Russia and China. In China, it is common in North China and Eastern Qinghai-Tibetan Plateau, inhabiting forests, scrubs, shrubs and grasslands on mountain slopes [42,43]. The axillary buds on the vertical stems may grow out and form stolons. The stolons usually take root on stolon nodes when reaching a moist substratum, and even a single stolon node can establish and grow as a ramet.

At the start of the experiment, fifteen plants of *F. orientalis*, each consisting of more than sixteen newly produced ramets (on stolons), were excavated around Maoxian Ecological Station, Chinese Academy of Sciences (31°41′07″N, 103°53′58″E; 1816 m asl.). The sampling site did not belong to part of any farms or national parks. *F. orientalis* is widespread in China and it is not an endangered or protected species, so we did not need any relevant permissions/permits for plant samples collection. Of 15 plants, each five plants were collected from a forest, shrubland and grassland situation, separately. In the same situation, each plant was collected at least 1000 m away from one another. They were thus considered as fifteen distinct genotypes [44]. The new ramets (first-year ramets) of these original plants were dissected into clonal fragments, each composed of two interconnected ramets of similar size. One ramet in each pair was referred to as the initial proximal part, indicating its relative proximity to the mother rosette, while the other as the initial distal part. With the stolon still intact between two ramets, these clonal fragments were planted in trays of sand for about three weeks. Once well established (rooted), the similar-size clonal fragments were chosen and transplanted into plastic pots (20 cm in diameter and 15 cm in height) filled with homogenized soil to a depth of 14 cm. The proximal and distal ramets of each clonal fragment were planted in a separate pot (forming a pair of pots), and they were connected by an intact stolon. In half of the pots the stolon was cut (severed treatment). Intact connection between paired ramets (intact stolon) allowed physiological integration between ramets, while in the severed treatments the stolon integration was impeded. Plants were grown in a glasshouse at Maoxian Ecological Station under a semi-controlled environment, with the day temperature range of 12-31°C and night temperature range of 9-15 °C, and the relative humidity range of 35-85%.

**Figure 1:**
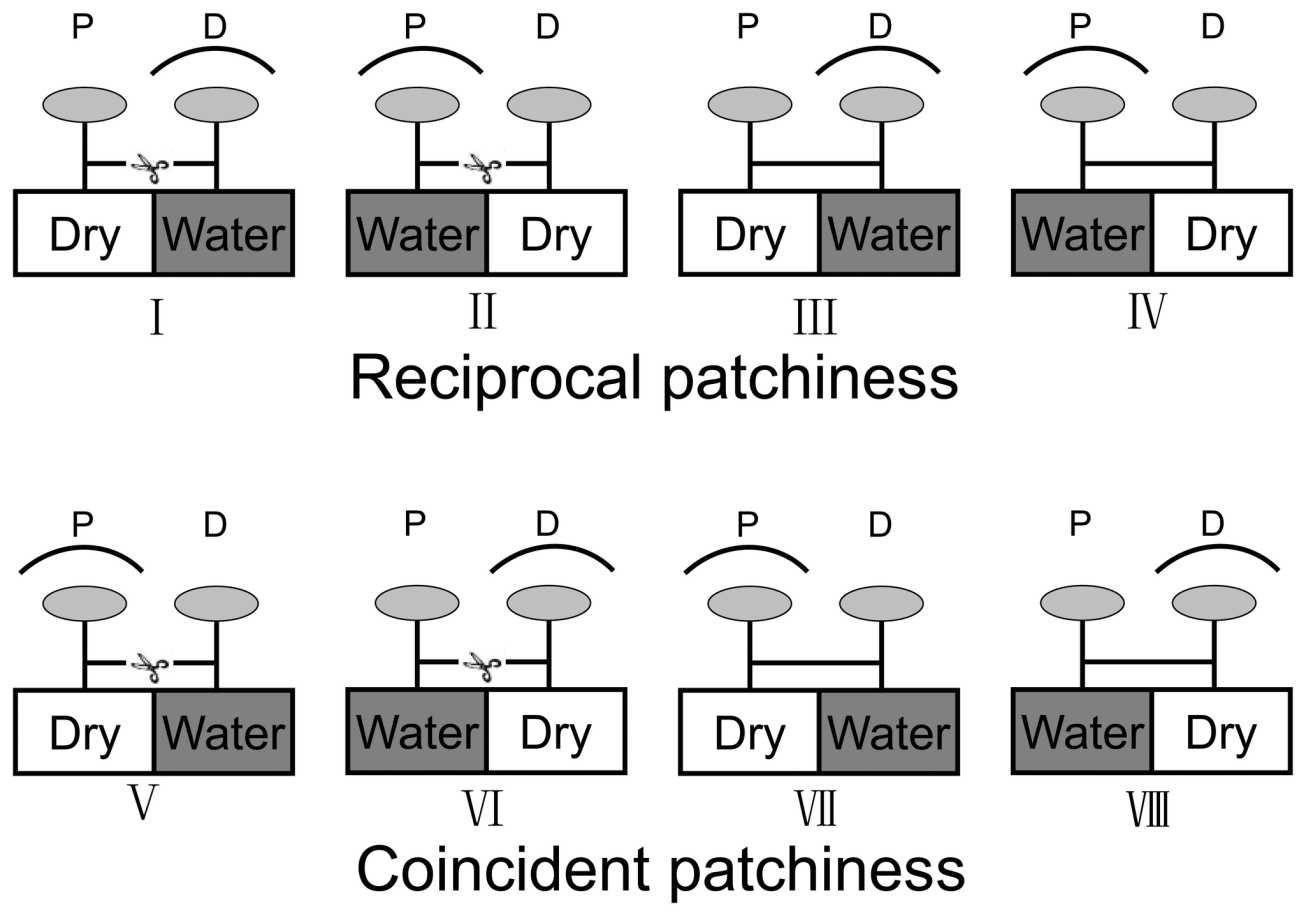
Experimental design. Reciprocal patchiness group: for severed pairs of ramets, the proximal ramets of clonal fragments (abbreviated as P in the figure) were given high light and low water treatment, and the distal ramets of the same clonal fragments (abbreviated as D in the figure) were given low light and high water treatment (I), or the proximal ramets of clonal fragments were given low light and high water treatment, and the distal ramets of the same clonal fragments were given high light and low water treatment (II); for intact pairs of ramets, the proximal ramets of clonal fragments were given high light and low water treatment, and the distal ramets of the same clonal fragments were given low light and high water treatment (III), or the proximal ramets of clonal fragments were given low light and high water treatment, and the distal ramets of the same clonal fragments were given high light and low water treatment (IV). Coincident patchiness group: for severed pairs of ramets, the proximal ramets of clonal fragments were given high light and low water treatment, and the distal ramets of the same clonal fragments were given low light and high water treatment (V), or the proximal ramets of clonal fragments were given low light and high water treatment, and the distal ramets of the same clonal fragments were given high light and low water treatment (VI); for intact pairs of ramets, the proximal ramets of clonal fragments were given high light and low water treatment, and the distal ramets of the same clonal fragments were given low light and high water treatment (VII), or the proximal ramets of clonal fragments were given low light and high water treatment, and the distal ramets of the same clonal fragments were given high light and low water treatment (VIII).

The experiment ran for 4 months, from 20 May to 20 September. At the beginning of the experiment, all ramets were about 2 cm tall. Both the intact and severed pairs of ramets were divided into two groups, and each group has four treatments (Figure 1). In each treatment, high light intensity corresponded to 100% photosynthetic photon flux intensity of the greenhouse daylight (the maximum light intensity is about 1600 μmol/m^2^/s) and low light intensity corresponded to approximately 15% of the high light intensity, which was achieved by covering the ramets with shading nets. High water availability was kept at 90% of field capacity (field capacity is 425g/kg) and low water availability was kept at 30% of field capacity. The pots were re-watered to their respective field capacity by replacing the amount of water transpired every second day. The amount of water was determined by weighing the pots. An empirical relationship between plant fresh weight (Y, g) and plant leaf area (X, cm^2^): Y = 0.096 X - 0.158 (R^2^ = 0.923, P<0.001) was used to correct pot water for changes in plant biomass. Additionally, 15 additional control pots were equipped with dead *F. orientalis* and the pots enclosed in plastic bags in the same way as other treatments. These pots were also weighed every second day in order to estimate evaporation from the soil surface. Evaporation from the soil surface was reduced by enclosing all pots in plastic bags sealed at the base of the stem of each ramet. A total of 8 g of slow-release fertilizer (13% N, 10% P and 14% K-Xinjin, Xinjin Compound Fertilizer Factory, Sichuan, China) was added to each pot during the experiment. 

### Measurements

After 4 months of treatment, the number of ramets was counted in the proximal and distal parts. Then, all parts of each plant in each pot were marked and harvested. Above- and belowground parts were separated in each pot and the biomass of each part was determined after drying at 70 °C for 48h. Finally, the ratio of root biomass to shoot biomass was derived for each half of the clonal fragments.

Costs and benefits of clonal integration were calculated separately for the proximal and distal parts in terms of biomass, number of ramets. Costs and benefits were defined as the difference in performance of the proximal and distal parts between intact and severed ramets [22,44-46]. 

To compare the costs and benefits between proximal and distal ramets and between reciprocal and coincident resources, the profit rate was calculated for the biomass of ramets or fragments as:

$$PR_{r}=\left(B_{ir}-B_{sr}\right)/B_{sr}$$ (1)

Where *PR*_*r*_ is the profit rate of ramets, *B*_*ir*_ is biomass of intact ramets and *B*_*sr*_ is biomass of severed ramets.

^*PR*^_*f*_^=(*B*^_*if*_^−*B*^_*sf*_^)/*B*^_*sf*_ (2)

Where *PR*_*f*_ is the profit rate of a fragment, *B*_*if*_ is biomass of an intact fragment and *B*_*sf*_ is biomass of a severed fragment.

### Statistical analysis

One-way ANOVAs with Tukey multiple tests were conducted to analyse the differences in the eight treatments. Three-way ANOVAs with types of patchiness (two levels: reciprocal and coincident), severance of stolon connection (two levels: severed and intact) and direction of resource transport (two levels: from proximal to distal ramet and from distal to proximal ramet) as fixed factors were carried out to test the differences in biomass, number of ramets and R/S ratio under reciprocal patchiness and coincident patchiness, respectively. T-test was conducted to analyse the differences of profit rate between treatments. All statistical analyses were done with the SPSS 18 for Windows statistical software package (IBM Corp., Somers, New York, USA). 

## Results

### Clonal growth

**Figure 2:**
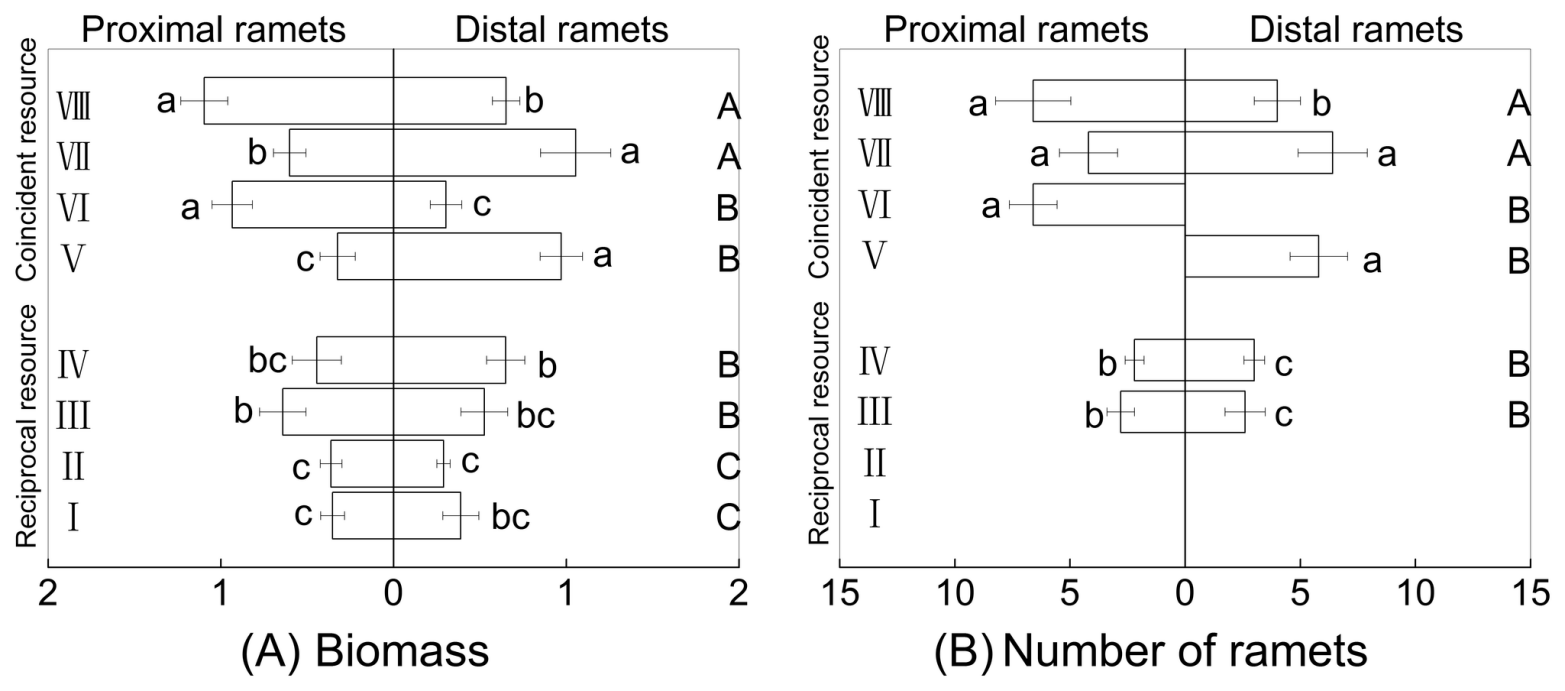
Biomass and number of ramets (Mean±S.E) of *Fragaria orientalis* under eight different treatments. The left horizontal bars represent the proximal part and the right horizontal bars represent the distal part. Biomass and number of ramets for whole clonal fragment is the sum of the proximal part and the distal part. For the proximal part and the distal part, separately, horizontal bars sharing the same lowercase letter are not significantly different at P=0.05. For the whole clonal fragment, horizontal bars sharing the same capital letter are not different at P=0.05. Treatments are coded as in Figure 1.

 When patchiness of above- and below-ground resources was reciprocal, intact clonal fragments of *F. orientalis* (III & IV) accumulated greater total biomass than those which were severed (I & II). Under high light and low water treatments both proximal and distal ramets of intact fragments had more biomass than those of severed fragments (IIIP vs. IP or IVD vs. IID). There were no significant differences between the biomass of intact ramets and that of severed ramets under low light and high water treatments (Figure 2A, Table 1). Both proximal and distal ramets of all intact fragments (III & IV) produced new ramets, while none were found from severed ramets (I & II) (Figure 2B, Table 1).

**Table 1:** F-values of three-way ANOVA which was used to test for the effects of types of patchiness (P), severing stolon (S), direction of resource transport (D) and their interaction (P*S), (P*D), (S*D), (P*S*D) on biomass, number of ramets and R/S in proximal ramets, distal ramets and clonal fragments.

Characters		d.f.	P	S	D	P*S	P*D	S*D	P*S*D
Proximal ramet	Biomass	1, 60	21.754^**^	10.041^**^	13.655^**^	0.066 ^ns^	25.094^**^	1.647 ^ns^	0.126 ^ns^
	No. of ramets	1, 60	40.918^**^	22.224^**^	18.543^**^	0.098 ^ns^	23.064^**^	5.777^*^	3.638 ^ns^
	R/S	1, 60	9.822^**^	0.336^ns^	13.187^**^	0.651 ^ns^	4.867^*^	23.115^**^	23.557^**^
Distal ramet	Biomass	1, 60	14.585^**^	9.972^**^	12.267^**^	0.013 ^ns^	13.316^**^	2.268 ^ns^	0.004 ^ns^
	No. of ramets	1, 60	33.535^**^	33.052^**^	16.983^**^	0.308 ^ns^	21.912^**^	4.949^*^	2.771 ^ns^
	R/S	1, 60	5.909^*^	0.352 ^ns^	10.337^**^	0.351 ^ns^	1.260 ^ns^	21.965^**^	15.844^**^
Fragment	Biomass	1, 60	34.325^**^	14.041^**^	15.415^**^	0.226 ^ns^	22.044^**^	2.668 ^ns^	0.826 ^ns^
	No. of ramets	1, 60	50.936^**^	21.225^**^	22.51^**^	0.378 ^ns^	21.063^**^	5.212^*^	2.694^ns^
	R/S	1, 60	12.432^**^	10.336^**^	15.127^**^	0.991 ^ns^	4.972^*^	24.144^**^	19.584^**^

Significance level: ns P > 0.05, * P < 0.05, ** P < 0.01

When the resource patchiness was coincident, intact clonal fragments of *F. orientalis* (VII & VIII) had more biomass than severed fragments (V & VI). Both proximal and distal ramets of intact fragments had more biomass than severed ramets under low light and low water treatments, while under high light and high water treatments (Figure 2A, Table 1), the biomass levels did not show any significant difference between intact and severed ramets. Both severed and intact ramets produced new ramets under high light and high water treatment but under conditions of low light and low water treatment (Figure 2B, Table 1), intact ramets produced new ramets whereas severed ramets did not.

### R/S ratio

**Figure 3:**
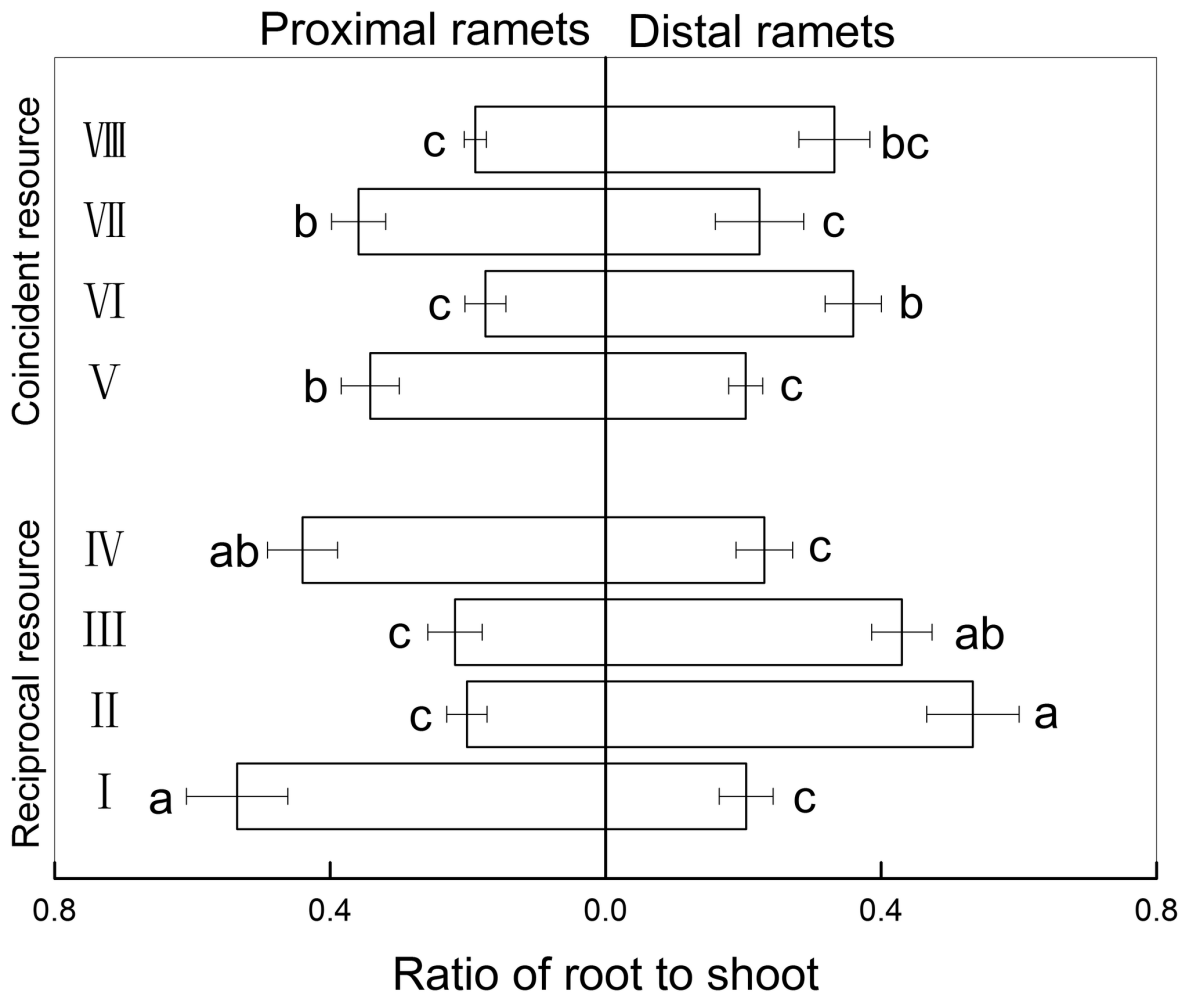
Ratio of root to shoot (Mean±S.E) of *Fragaria orientalis* under eight different treatments. The dotted and open horizontal bars represent the proximal part and the distal part. For the proximal part and the distal part, separately, horizontal bars sharing the same lowercase letter are not significantly different at P=0.05. Treatments are coded as in Figure 1.

When patchiness of above- and below-ground resources was reciprocal, intact ramets in low light and high water treatments had higher R/S ratio than severed ramets. Intact ramets in high light and low water treatments had lower R/S ratio than severed ramets (Figure 3, Table 1). When the pattern of resource patchiness was coincident, there was no significant difference in R/S ratio between intact and severed ramets (Figure 3, Table 1).

### Cost-benefit analysis

**Figure 4:**
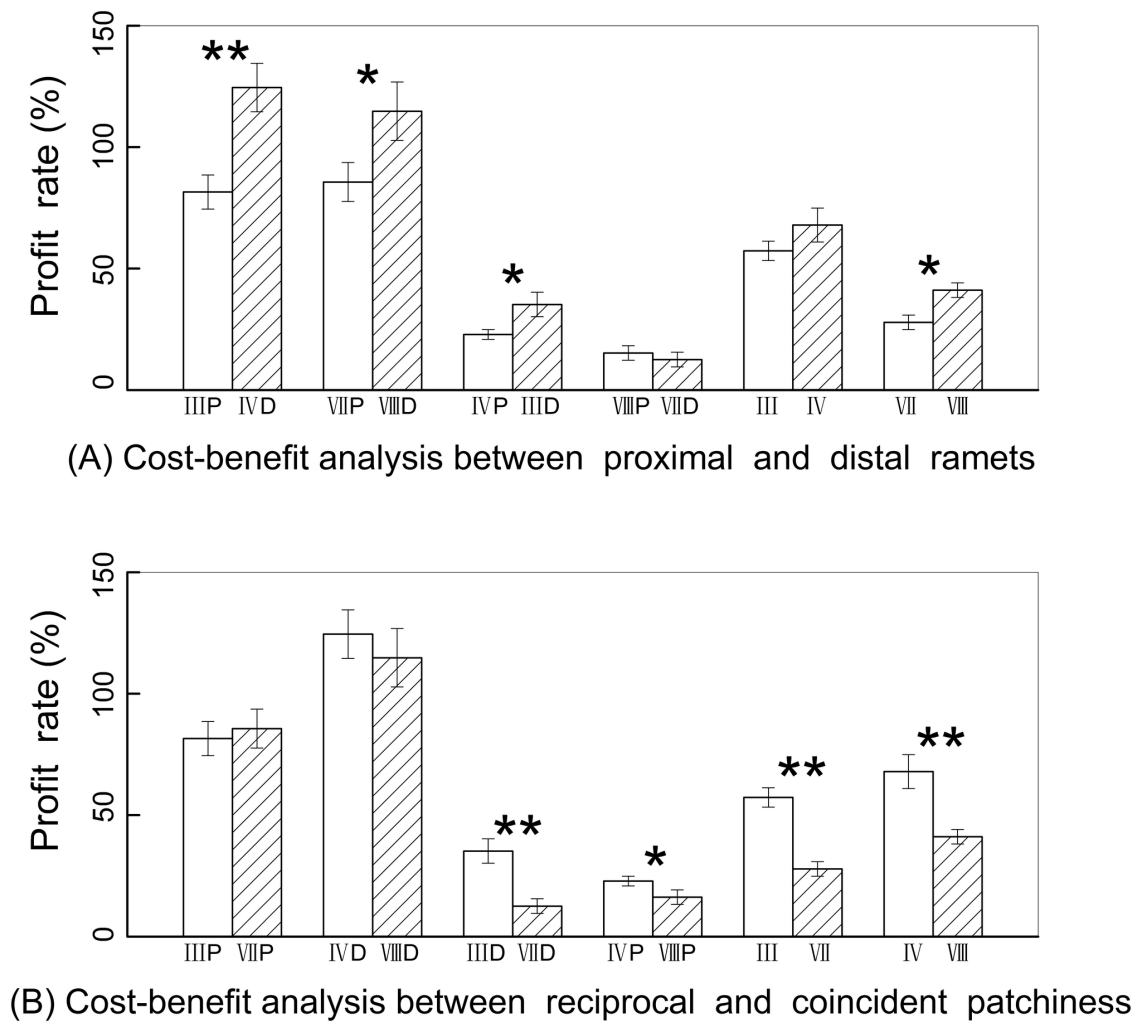
Cost-benefit analysis. The profit rate of ramets and fragment were calculated based on formula (1) and formula (2) separately. Significance level: * P < 0.05, ** P < 0.01. Treatments are coded as in Figure 1.

The resource-scarcity ramets (scarcity of water, light or both) obtained more profit when benefiting ramets were distal ramets than when benefiting ramets were proximal ramets in both reciprocal and coincident patchiness, while profit rate of ramets in favourable conditions (high-water and high-light treatment) showed no significant difference whether the target ramets were distal or proximal ramets in coincident patchiness (Figure 4A). In reciprocal patchiness, the profit rate of fragments showed no significant difference between distal ramets and proximal ramets when both were benefit ramets (Figure 4A). In coincident patchiness, the profit rate of fragments was higher when benefiting ramets were distal ramets rather than when they were proximal ramets (Figure 4A). The profit rate of ramets under low water treatment showed no difference between reciprocal and coincident patchiness (Figure 4B). The profit rate of fragments as well as that of ramets under high water treatment was higher in reciprocal patchiness than in coincident patchiness (Figure 4B).

## Discussion

The results partly supported the hypothesis that effects of clonal integration on the performance of clonal plants, as measured by accumulation of biomass, production of new ramets and R/S ratio, are dependent on whether connected ramets experience reciprocal or coincident patchiness of above- and below-ground resources. 

The high clonal integration had been found in previous studies on *F. orientalis* [22,30,47] and other *Fragaria* species [20,48-50]. In this study, clonal integration between the ramets of intact clonal fragments was observed both in reciprocal patchiness and in coincident patchiness by comparison with severed fragments. In coincident patchiness, resources were transported only from ramets in high light and high water to ramets in low light and low water. The ramets growing in favourable habitats may nurse those in unfavourable habitats. These results were consistent with studies on clonal integration between ramets in heterogeneous resource availability, although these studies were not on coincident patchiness [22,24,44,51,52]. The findings further supported the source-sink hypothesis, suggesting that differences in resource supply drive the sharing process, with resources moving from ramets with high access to resources to those with low access to resources. In reciprocal patchiness, the share of resources between the intact ramets was bidirectional and *F. orientalis* developed division of labour in response to a heterogeneous environment, as measured by R/S ratio. Such division of labour between connected clonal ramets has been reported in many previous studies [37-40]. These studies clearly showed the high potential benefits of division of labour to enhance resource capture of clonal plants and thereby to increase their performance in heterogeneous habitats compared to non-clonal plants. The difference of integration mode between ramets in the two heterogeneous habitats mentioned above might be a possible explanation for the different effects of reciprocal and coincident patchiness on the performance of *F. orientalis*.

Another possible explanation for different effects of reciprocal and coincident patchiness on performance might be the degree of physiological or morphological plasticity. Phenotypic plasticity is the ability of a genotype to modify its growth and development in response to changes in environmental conditions [53,54]. For plants, the well-developed plasticity of many traits is usually interpreted as an adaptive response to environmental heterogeneity as a consequence of immobility and modular growth [55]. Such high plasticity has already been observed in many clonal plants [56-58]. This high plasticity response to resource heterogeneity may induce the different effects of reciprocal and coincident patchiness on performance. The plasticity of clonal plants can be modified by clonal integration [59]. So, different effects of reciprocal and coincident patchiness on performance might be the combined effect of both physiological integration and phenotypic plasticity.

These findings add greatly to our knowledge of the patterns of resource heterogeneity which modify the growth of clonal plants. It is already known that the size of resource patches and the difference between the levels of a resource in different patches can affect the growth of clonal plants [21,39]. The results from this study show that whether high levels of two resources occur in the same patches (coincident patchiness) or in different patches (reciprocal patchiness) can modify the performance of clones. 

 We found that, both in reciprocal and coincident patchiness, ramets in unfavourable patches had a higher profit rate when the benefiting ramets were distal. One possible explanation is that the source-sink driving force of clonal integration in our experiment came from both resource difference and age difference. Resources translocate from ramets with high resource levels to those with low resource levels under the driving force of resource difference [31,45,51,60]. Resources also translocate from old ramets to young ramets under the driving force of age [29,48,61,62]. When both of these driving forces work in the same direction, the beneficial ramets will get most benefit, whereas when the driving forces work in opposing directions, the beneficial ramets will benefit less. It also can be inferred indirectly from these results that, in coincident patchiness, the profit rate of ramets in favourable patches were not affected by integration direction because they did not need to input resources from connected ramets. A well-documented example of the benefits from clonal integration being affected by constraints on the translocational direction of resource, is that movement of water and assimilated carbon are mainly acropetal [63-66], whereas some studies showed they are bidirectional [67,68]. In this study, it seems possible that movement of water and assimilated carbon were bidirectional, but that it was easier to move from proximal ramets to distal ramets than from distal ramets to proximal ramets. Constraints on translocation of resource may be an explanation for the differential effects of reciprocal and coincident patchiness on performance.

As for the profit rate of the whole clonal fragment, when in reciprocal patchiness, the biomass did not show significant difference between distal and proximal ramets when they are beneficial ramets. While in coincident patchiness, the profit rate of the clonal fragment with distal ramets as the beneficial ramets was significantly higher than that when proximal ramets were beneficial ramets. The reason is not clear but a possible interpretation is that importation of photosynthate to ramets in low light may be enhanced if they were accompanied by water importation, and movement of water and photosynthate was partly acropetal. Similar results have also been observed in *Cynodon dactylon* [34]. This can be considered as another explanation for differential effects of reciprocal and coincident patchiness on performance. 

As for profit rate between reciprocal and coincident patchiness, the profit rate of ramets under low water treatments showed no difference between in reciprocal and coincident patchiness, while the profit rate of fragments, as well as that of ramets under high water treatments, were higher in reciprocal patchiness than in coincident patchiness. This can be explained by the fact that only ramets in unfavourable patches (ramets in low light and low water treatment) can accumulate more biomass than severed ramets in coincident patchiness, but ramets in favourable patches did not get significant benefit and showed no difference from severed ramets. However, they were only in one unfavourable condition (low light or low water), their connected ramets can accumulate more biomass than severed fragments in reciprocal patchiness. So difference of profit rate of fragments between reciprocal and coincident patchiness was mainly caused by different profit rate of high-water ramets between the two types of heterogeneous patchiness. 

Although clonal fragments of *F. orientalis* obtained a higher rate of benefit in reciprocal patchiness than in coincident patchiness; biomass and number of ramets of clonal fragments were larger in coincident patchiness than those in reciprocal patchiness, which was not what we expected. Thus, coincident patchiness is better for growth of *F. orientalis* in respect of biomass and clonal reproduction. This growth difference under the same total resource in two types of heterogeneous patchiness perhaps can be explained by constraints of clonal integration. Specialization and cooperation between ramets of clonal plants represent highly complex interactions between plants and their environments, which involve many processes [38]. Each process has the potential to constrain the viability and the profitability of clonal integration. This constraint was particularly acute in reciprocal patchiness. The exchange of assimilates and nutrients, for instance, can be constrained by the absence of a water potential gradient running parallel to environmental gradients in nutrient supply [4,69]. Therefore, resource acquisition of *F. orientalis* in reciprocal patchiness may be more influenced by the state and direction of integration, and the clonal fragment had less biomass and ramets because of resource limitation. While in coincident patchiness, *F. orientalis* in favourable patchiness can get what it needed without the limit of integration, and it can spread to unfavourable habitats based on the well-developed ramets from favourable habitats [70]. Although clonal plants developed effective strategies to adapt their habitats, at least in this study, they can’t offset the effect of their habitats.
